# Development and Validation of an LC-MS/MS Method for the Quantification of Methenamine in Raw Milk and Bovine Muscle and Its Application to Incurred Samples

**DOI:** 10.3390/molecules30244807

**Published:** 2025-12-17

**Authors:** Sunjin Park, Chung-Oui Hong, Se-Hyung Kim, Seon-Young Lee, Inhae Jeon, Do Hui Kim, Hyun-Ok Ku, Mi-Young Park

**Affiliations:** Veterinary Drugs & Biologics Division, Animal and Plant Quarantine Agency (APQA), 177, Hyeoksin 8-ro, Gimcheon-si 39660, Gyeongsangbuk-do, Republic of Korea; bluedonald@korea.kr (S.P.); hco1207@korea.kr (C.-O.H.); sehyungkim@korea.kr (S.-H.K.); fghjk1010@korea.kr (S.-Y.L.); inhae2684@korea.kr (I.J.); kdh0427@korea.kr (D.H.K.); kuho@korea.kr (H.-O.K.)

**Keywords:** methenamine, LC–MS/MS, raw milk, bovine muscle, residue analysis, Positive List System (PLS)

## Abstract

Methenamine, a urinary antiseptic with antimicrobial properties, decomposes into toxic formaldehyde under acidic conditions. Its use is prohibited in dairy cattle in Korea to prevent harmful residues in milk. This study was designed to develop and validate a sensitive and reliable LC–MS/MS method for determining methenamine in raw milk and bovine muscle in compliance with the Positive List System (PLS) regulations. Samples were extracted with acetonitrile (ACN)–methanol (MeOH) (7:3, *v*/*v*) containing ammonia water, followed by defatting with n-hexane and purification with primary secondary amine (PSA). Chromatographic separation was performed on a hydrophilic interaction liquid chromatography (HILIC) column, and quantification was conducted using matrix-matched calibration to minimize matrix effects. The method showed excellent linearity (R^2^ > 0.999), low limits of quantification (LOQ) (0.49 μg/kg for raw milk; 0.64 μg/kg for bovine muscle), and acceptable recoveries (78.1–102.8%) with precision (CV ≤ 8.75%), meeting Codex CAC/GL 71-2009 criteria. Stability studies demonstrated that methenamine remained stable in stock solutions, working standards and processed extracts under the storage and handling conditions used. Application to incurred samples resulted in the detection of methenamine in 2 of 32 raw milk samples (0.65 and 1.14 μg/kg) but in none of the 25 bovine muscle samples, with all detected levels below the Korean PLS limit. These findings confirm that the developed method is accurate, sensitive, and applicable for routine surveillance of methenamine residues to ensure consumer safety.

## 1. Introduction

Methenamine is a drug widely used in the treatment of urinary tract infections and nephropathy. Under acidic conditions, it decomposes into formaldehyde and ammonia, and the resulting formaldehyde exhibits strong antimicrobial activity [1,2]. It has been employed in veterinary practice for the treatment of chronic inflammatory diseases such as endometritis, cystitis, and pyelonephritis in cattle and pigs, as well as for treatment of coccidiosis in poultry [3]. In Europe, methenamine is also used as a preservative in animal feed, particularly in silage, to improve fermentation quality [4]. Moreover, it is approved as a food and feed additive under specific conditions, such as in the production of provolone cheese, where it is permitted at a level corresponding to 25 mg/kg of formaldehyde to inhibit gas-producing *Clostridia* bacteria [5].

However, formaldehyde has been reported to cause carcinogenic effects as well as both acute and chronic toxicity [2]. As a result, methenamine is not approved as a food additive in countries such as the United States, Russia, China, Australia, and New Zealand [6]. This raises safety concerns not only regarding its use as a food additive, but also when applied as a veterinary drug or feed additive. In particular, if methenamine administered to food-producing animals remains as a residue in edible animal products, it may pose risks to human health, including antibiotic resistance and allergic reactions. Therefore, considering the potential health risks associated with formaldehyde formation, systematic monitoring of methenamine residues is required.

In Korea, a maximum residue limit (MRL) of 0.01 mg/kg has been established for methenamine in major livestock species, including cattle, pigs, and chickens. However, its use in dairy cattle has not been approved due to the lack of residue evaluation in milk. Furthermore, with the implementation of the Positive List System (PLS) in January 2024, a uniform MRL of 0.01 mg/kg has been applied to all unapproved veterinary drugs. As a result, substances such as methenamine, which are approved for use in cattle but not in dairy cows, now require stricter and more systematic regulation. Therefore, it is essential to develop a reliable analytical method capable of detecting methenamine residues in raw milk and bovine muscle.

Previous studies have mainly focused on the simultaneous analysis of methenamine along with various other food additives in dairy products, such as cheese and milk, where it is used as a preservative, particularly in Europe. For example, LC-MS/MS has been used to detect multiple preservatives, including methenamine, in cheese [7,8], and UHPLC/ESI Q-Orbitrap mass spectrometry combined with QuEChERS extraction has been applied to dairy products such as milk and yogurt [8]. Similar multi-residue methods have also been developed for meat and fish products [9]. However, these studies mainly focused on multi-residue analysis, which often resulted in relatively high limits of quantification (LOQs) for methenamine [7,9]. Moreover, some used extraction solvents containing acidic components that could induce methenamine degradation, without consideration of its chemical stability [8,9]. In several cases, C_18_ columns, which are not suitable for separating highly polar compounds such as methenamine, were also employed [7,8].

Targeted analytical methods for methenamine have also been established in various matrices. In dairy products, a GC-MS/MS method using cation-exchange solid-phase extraction (SPE) and an isotope-labeled internal standard was reported [10], and HPLC-based methods have been used for the processed foods such as noodles, cheese, and tofu snacks [11,12]. LC-MS/MS has been applied for toxicokinetic studies in plasma [13]. In animal-derived foods, LC-MS/MS method combined with an internal standard and a modified QuEChERS procedure was reported for porcine muscle, kidney, and liver [14]; a selective extraction method using molecularly imprinted polymers (MIPs) was developed for eggs [6]; and a MIPSPE-GC-MS/MS method was applied to milk [15]. The official analytical method issued by the Ministry of Food and Drug Safety (MFDS) of Korea [16] also describes an LC-MS/MS method based on the QuEChERS approach for livestock products. These single-analyte methods generally used acetonitrile (ACN) as the extraction solvent [6,14,15,16], and either primary secondary amine (PSA)-based QuEChERS [14,16] or MIP-based purification techniques [6,15]. Matrix effects were addressed by employing internal standards [14] or matrix-matched calibration curves [16]. However, in most of these studies, matrix-matched calibration curves were prepared by spiking blank matrices with standard solutions before pretreatment [6,14,15,16]. As a result, recoveries exceeding 80% were frequently reported, but these values did not necessarily reflect the absolute extraction efficiency of methenamine in ACN-based solvents.

Under the PLS, a more reliable and efficient approach to residue analysis requires, first, maximizing the absolute extraction efficiency; second, removing interfering substances without loss of the analyte; and third, minimizing matrix effect during LC-MS/MS quantification. Methenamine (hexamethylenetetramine) is a small, highly polar compound that is freely soluble in water and prone to acid-catalyzed hydrolysis. These characteristics make conventional reversed-phase LC analysis difficult and require carefully controlled extraction conditions to prevent degradation and ensure adequate retention.

Therefore, in this study, we aimed to develop a rapid and robust analytical method for the determination of methenamine in raw milk and bovine muscle, using readily available laboratory reagents and the QuEChERS technique, instead of costly isotope-labeled internal standards or MIPs. In our study, all validation parameters, including recovery, were evaluated using matrix-matched calibration curves prepared by adding standard solutions to pretreated blank matrices. This study also aims to ensure the reliability and efficiency of the method through validation, and to assess its applicability to the analysis of residues in livestock-derived food products.

## 2. Results and Discussion

### 2.1. Optimization of Sample Pretreatment

#### 2.1.1. Comparative Evaluation with MFDS Method

To evaluate the recovery of methenamine from raw milk and bovine muscle, the MFDS method (Table 1) [16] was applied as a reference. The method employs ACN containing 10 mM ammonium formate as the extraction solvent, followed by purification with n-hexane, magnesium sulfate (MgSO_4_), and PSA prior to LC-MS/MS analysis, but it showed low recovery rates (19.9% for raw milk and 23.4–26.1% for bovine muscle) [16]. These findings underscored the need for improved sample pretreatment. Accordingly, optimization of the extraction and purification procedures was first performed using raw milk as a model matrix, and the refined conditions were subsequently applied to bovine muscle. Various extraction solvents and purification parameters were tested to enhance the removal of fat and other interfering substances, and the corresponding recoveries were compared. The primary criterion for evaluating recovery was the calibration curve, constructed using a matrix-matched approach. In each experimental protocol, blank samples were processed accordingly, spiked with standard solutions at defined concentrations, and analyzed to obtain the calibration curve. Since matrix composition can vary depending on the pretreatment method, a new matrix-matched calibration curve was established for each condition to ensure accurate recovery assessment.

#### 2.1.2. Optimization of Purification

To improve the recovery of methenamine in raw milk by effectively removing lipids, three fat elimination methods were compared, while keeping the extraction solvent (ACN) and the purification step (MgSO_4_ + PSA) constant: C_18_ sorbent (500 mg) [17,18], addition of n-hexane [14], and freeze-out [19,20]. C_18_ sorbent is widely used to eliminate long-chain fatty compounds and other non-polar interferences [18], whereas n-hexane effectively dissolves lipophilic substances such as fat. The freeze-out method was also tested to assess its ability to remove both fat and other ACN-insoluble interferences, with the expectation that purification could be achieved through centrifugation [19,21].

Although the freeze-out method yielded the highest recovery (41%) among the compared approaches, its effectiveness in fat removal was limited. Animal fat typically solidifies upon freezing, allowing separation by centrifugation. However, raw milk contains only approximately 3.7% fat, far less than that of meat products (e.g., 5–30% in bovine). Moreover, because milk fat exists in an oil-in-water emulsion [22], freezing at −70 °C for 20 min was insufficient for effective separation, and the freeze-out method was excluded from the procedure. Meanwhile, the recoveries with C_18_ and n-hexane were 23.7% and 31.8%, respectively. The lower recovery with C_18_ was attributed to the adsorption of methenamine through hydrogen bonding between its amine group and silanol groups on the sorbent surface, as reported by Xu et al. [14]. Ultimately, n-hexane addition was selected as the optimal fat-removal method due to its superior efficiency in eliminating lipids and other matrix interferences.

Purification conditions were further optimized to remove additional interferences in raw milk, including moisture, organic acids, sugars, and fatty acids. With the extraction solvent (ACN) and the fat-removal step (n-hexane) fixed, three purification approaches were compared: PSA alone, PSA with Na_2_SO_4_, and PSA with MgSO_4_. The highest recovery (76.7%) was obtained with PSA alone, whereas recoveries decreased to 54.5% with Na_2_SO_4_ and 31.8% with MgSO_4_. The lower recovery observed with MgSO_4_ was likely due to the adsorption of methenamine onto Mg^2+^, consistent with previous findings [14], likely involving coordination between the amine group of methenamine and Mg^2+^ ions [23]. Although Na_2_SO_4_ and MgSO_4_ are commonly used to remove residual moisture and shorten evaporation time, PSA alone was selected as the final purification sorbent because it provided higher recovery. PSA is particularly effective in removing matrix components such as polar organic acids, pigments, sugars, and fatty acids [8].

#### 2.1.3. Optimization of Extraction

The extraction procedure was systematically optimized based on the physicochemical characteristics of methenamine, a highly polar and water-soluble compound (Log P = −2.84) that undergoes acid-catalyzed hydrolysis (pKa = 4.89). Initially, the MFDS method using ACN with ammonium formate was evaluated in raw milk, but methenamine showed poor recovery (19.9%), indicating that a salt-containing extraction system was unsuitable. Removing the salt and using ACN alone improved recovery of 31.8%, suggesting that salting-out conditions hinder transfer of this highly polar analyte into the organic phase. Therefore, all salts and acidic additives were excluded from the subsequent optimization, and a mildly basic environment was introduced to stabilize methenamine during extraction.

**Figure 1:**
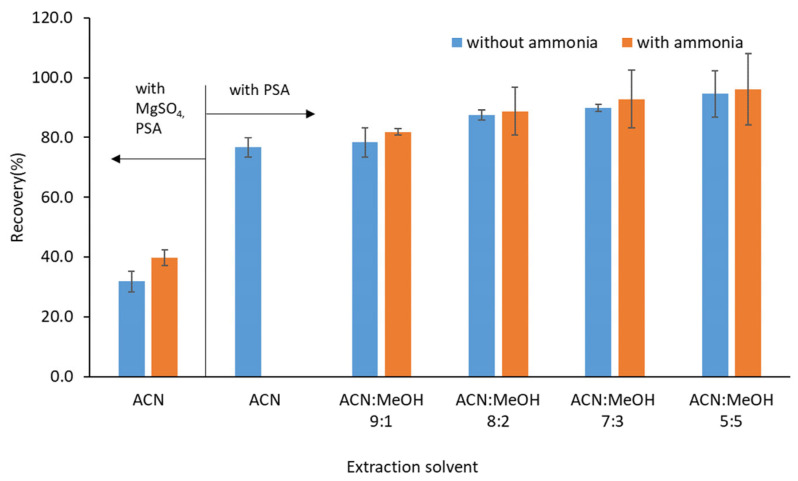
Influence of the composition of extraction solvent on the recovery of methenamine in raw milk spiked at 20 µg/kg (using defatting with hexane).

Using ACN extraction followed by n-hexane defatting and PSA cleanup, methenamine recovery reached 76.7%. To further improve extraction efficiency, alternative solvent systems were investigated. Following previous approaches of Fuselli et al. [7] and Molognoni et al. [9], extraction was performed using varying ratios of ACN and methanol (MeOH), with and without ammonia water. Increasing the proportion of MeOH enhanced recovery, and the addition of ammonia further improved extraction efficiency (Figure 1). This effect is consistent with the higher polarity of MeOH (Log P = −0.74) relative to ACN (Log P = −0.34), which increases the solubility of highly polar methenamine. However, excessive MeOH led to the co-extraction of matrix components, significantly prolonging the evaporation step. Therefore, an ACN:MeOH ratio of 7:3 (*v*/*v*) was selected as the optimal compromise between recovery, matrix cleanliness, and workflow efficiency.

The pH of the extraction medium was also critical for analyte stability. Because methenamine readily undergoes acid-catalyzed hydrolysis, maintaining mildly basic conditions helps prevent degradation and minimize interactions with acidic matrix components. In preliminary trials, the use of a large volume of dilute ammonium hydroxide (NH_4_OH) increased the aqueous phase volume and markedly prolonged nitrogen evaporation without providing any clear improvement in recovery or precision. Therefore, a small volume of concentrated ammonium hydroxide solution (28~30% NH_3_) was added directly to the ACN:MeOH (7:3, *v*/*v*) mixture. This approach provided sufficient basicity to stabilize methenamine without substantially increasing the aqueous volume or evaporation time. The final extraction procedure thus consisted of ACN:MeOH (7:3, *v*/*v*) containing a small amount of concentrated NH_4_OH, followed by n-hexane defatting and PSA clean-up. This optimized protocol yielded recoveries within the validation criteria for both raw milk and bovine muscle, with acceptable intra- and inter-day precision, demonstrating its suitability for routine residue analysis.

### 2.2. LC Method Development

Preliminary trials on conventional reversed-phase C_18_ columns resulted in negligible retention and poor peak shapes. Therefore, a HILIC column was selected to provide stronger retention and improved separation for highly polar and ionic analytes.

The mobile phase was optimized to achieve stable retention, symmetrical peak shape, and robust ionization in positive electrospray ionization (ESI) mode. Mobile phase A consisted of water containing 5 mM ammonium acetate and 0.1% formic acid, which served as a volatile buffering system to stabilize pH, enhance protonation, and improve ionization consistency. Mobile phase B was 100% ACN, providing the high-organic environment required for effective HILIC separation.

The chromatographic system was operated in isocratic mode at an A:B ratio of 25:75 for 10 min. Maintaining this high ACN proportion was essential to preserve the HILIC partitioning mechanism and achieve sufficient retention of the highly polar analyte. Under these conditions, a sharp and symmetrical peak, stable baseline, and high reproducibility were obtained for both matrices. Gradient elution programs were examined but did not improve separation performance and instead introduced unnecessary complexity and potential variability in retention time. Therefore, the simple isocratic HILIC condition with a 10 min run time was adopted as the final LC method for methenamine determination in raw milk and bovine muscle.

### 2.3. Validation of Method

#### 2.3.1. Matrix Effect

In LC-MS/MS analysis, the matrix effect refers to the influence of coexisting sample components on the ionization of the target analyte, thereby affecting the accuracy of quantification. It is a critical factor that must be considered in LC-MS/MS-based methods [24]. In this study, the extent of the matrix effect (ME) was evaluated by comparing the slopes of matrix-matched calibration curves with those of solvent-based calibration curves.

ME (%) = (*Slope _matrix-matched standard curve_/Slope _solvent standard curve_* − 1) × 100 (1)

Matrix effects can manifest as either a positive effect, enhancing ionization efficiency, or a negative effect, suppressing it [24]. Their magnitude depends on various factors, including the nature of the sample matrix, pretreatment procedures, chromatographic conditions, mobile phase additives, and the ionization technique used. In particular, ESI is generally more susceptible to matrix effects than atmospheric pressure chemical ionization (APCI) [25,26].

In this study, methenamine showed strong ion suppression, with values of −84.7% in raw milk and −97.9% in bovine muscle. To compensate for these effects—commonly addressed by various strategies in previous studies [25,27,28,29]—matrix-matched calibration curves were employed to ensure accurate and reliable quantification.

#### 2.3.2. Linearity, Limits of Detection (LOD) and Limits of Quantification (LOQ)

The calibration range for the matrix-matched curves was established according to the CODEX CAC/GL 71-2009 guidelines [30]. Linearity was assessed using matrix-matched calibration curves prepared for both raw milk and bovine muscle. The full chromatograms, including all matrix components and background signals within the entire retention window, are provided in the Supplementary Materials (Figure S1) to demonstrate the absence of interfering peaks near the analyte. In addition, the complete calibration curves for both matrices are presented in the Supplementary Materials (Figure S2). Each calibration level represents the mean peak area and standard deviation obtained from analyses conducted over three consecutive days, confirming the consistency and reproducibility of the instrumental response. In addition, correlation coefficients (R^2^) exceeding 0.999 were obtained for both raw milk and bovine muscle, indicating excellent linearity.

The LOD and LOQ were determined based on the signal-to-noise (S/N) approach and the statistical calculation using the standard deviation of the residuals (σ) and the slope (S) of the calibration curve. The equations used were:

LOD = 3.3σ/S, LOQ = 10σ/S(2)

The LOD and LOQ calculated statistically were 0.16 and 0.49 μg/kg for raw milk, and 0.21 and 0.64 μg/kg for bovine muscle, respectively. S/N ratios were evaluated to experimentally confirm the statistically calculated LOD and LOQ. To ensure an accurate measurement of noise, the S/N ratios were calculated using the baseline region immediately adjacent to the analyte retention time. At the LOD-level concentration of 0.4 μg/kg, the S/N ratios were 8 for raw milk and 7.4 for bovine muscle. At the LOQ-level concentration of 0.8 μg/kg, S/N ratios were 14.6 and 13.1, respectively. The method’s sensitivity fully met the domestic PLS criterion of 10 μg/kg. Furthermore, its LOQ was lower than that of the MFDS method (2 μg/kg), confirming the improved sensitivity and analytical performance of the method.

The concentration of methenamine in samples was calculated as follows:

C_S_ = C_C_ × V/m (3)

where C_S_ is the methenamine concentration in the sample (µg/kg), C_C_ is the methenamine concentration determined from the calibration curve (ng/mL), V is the final volume (mL), and m is the sample weight (g).

#### 2.3.3. Accuracy and Precision

Accuracy and precision were evaluated at concentrations of 1, 10, and 40 µg/kg for raw milk, and 5, 10, and 40 µg/kg for bovine muscle. Accuracy was evaluated by calculating the mean recovery after spiking blank samples with standard solutions, based on the matrix-matched calibration curve prepared by adding standard solutions to pretreated blank matrices. The resulting recoveries ranged from 78.1% to 102.8%, satisfying the acceptance criteria of 70–120% specified in CAC/GL 71-2009 [30] (Table 2). Precision was assessed at the same concentrations, based on intra-day and inter-day variability. Intra-day precision was determined from 6 replicates per concentration within a single day, while inter-day precision was assessed from 12 replicates per concentration over 3 consecutive days. As shown in Table 2, the coefficients of variation (CV) were ≤7.64% for intra-day and ≤8.75% for inter-day, indicating good precision.

#### 2.3.4. Stability Tests

Stability of methenamine in raw milk and bovine muscle was evaluated under short-term, freeze–thaw and autosampler conditions using QC samples at two concentration levels (low and high) following internationally accepted bioanalytical validation guidelines [31,32]. For each stability test, the mean concentration measured after the stability challenge was compared with that of freshly prepared QC samples (time-zero), and the relative error (% RE) was calculated.

Across all stability conditions and both matrices, the %RE values remained within the ±15% at both QC levels, with no systematic trends suggesting degradation or analyte loss. These results confirm that methenamine is stable during short-term handling, frozen storage, repeated freeze–thaw cycles, and autosampler waiting periods. Therefore, no stability-related bias is expected during routine analysis, supporting the reliability of the validated LC-MS/MS method for both raw milk and bovine muscle. The numerical stability data for each matrix, concentration level, and condition are summarized in Table 3.

### 2.4. Application to Incurred Samples

**Figure 2:**
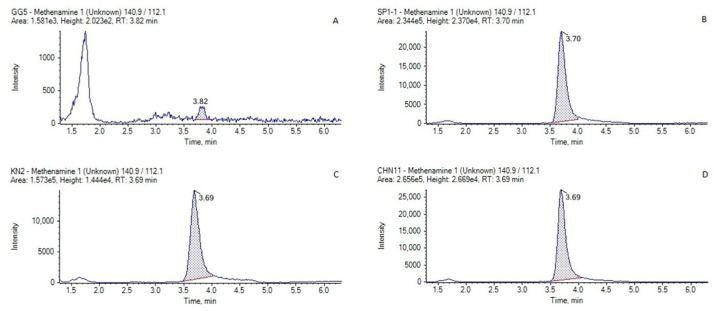
Chromatograms of the following raw milk samples. (**A**) blank raw milk, (**B**) blank raw milk spiked with methenamine at 1 μg/kg, (**C**) sample detected at 0.65 μg/kg, (**D**) sample detected at 1.14 μg/kg.

A total of 32 raw milk samples were collected from milk collection centers in 7 regions across the country, and 25 bovine muscle samples were obtained from slaughterhouses in 8 regions. All samples were stored at −20 °C until analysis. Quantification was performed using matrix-matched calibration curves over the concentration range of 0.4–100 ng/mL. For accuracy control, blank samples spiked with standard solutions were analyzed concurrently. Positive identification was based on retention time and ion ratio in accordance with CODEX CAC/GL 71-2009 guidelines [30]. As shown in Figure 2, methenamine was detected in two of the 32 raw milk samples at concentrations of 0.65 and 1.14 μg/kg, both above the LOQ but still below the PLS threshold. In contrast, no methenamine was detected in any of the 25 bovine muscle samples. The retention times of the positive samples were within ±0.2 min of the reference standard, and the ion ratios were within the allowable tolerance of ±25%, confirming the reliability of the developed method. These findings demonstrate that the method is suitable for the quantitative determination of methenamine in real samples.

### 2.5. Convenience and Economics of Proposed Method

The proposed method showed high practical applicability, as it employed readily available solvents and sorbents commonly used in laboratories, without requiring expensive isotope-labeled internal standards, MIPs [6], cation-exchange SPE [10], or magnetic SPE (M-SPE) [11]. The simplified procedure reduced overall cost and shortened the sample pretreatment time to approximately two-thirds of that required by the MFDS method, thereby improving analytical efficiency. In addition, the optimized extraction solvent and purification conditions minimized methenamine loss and maximized extraction efficiency, enabling accurate residue analysis. This is particularly relevant under the PLS, which requires highly sensitive detection near the LOQ. Characterized by its low LOQ, this LC-MS/MS-based method offers substantial practical value for the monitoring of veterinary drug residues in livestock products.

### 2.6. Greenness Assessment

The environmental impact of the developed analytical method was assessed using the AGREE (Analytical GREEnness) metric, which evaluates compliance with the 12 principles of Green Analytical Chemistry [33]. The assessment considered various aspects of the procedure, including sample amount, type and volume of solvents, number of preparation steps, waste generation, and the energy demand of the instrumentation.

**Figure 3:**
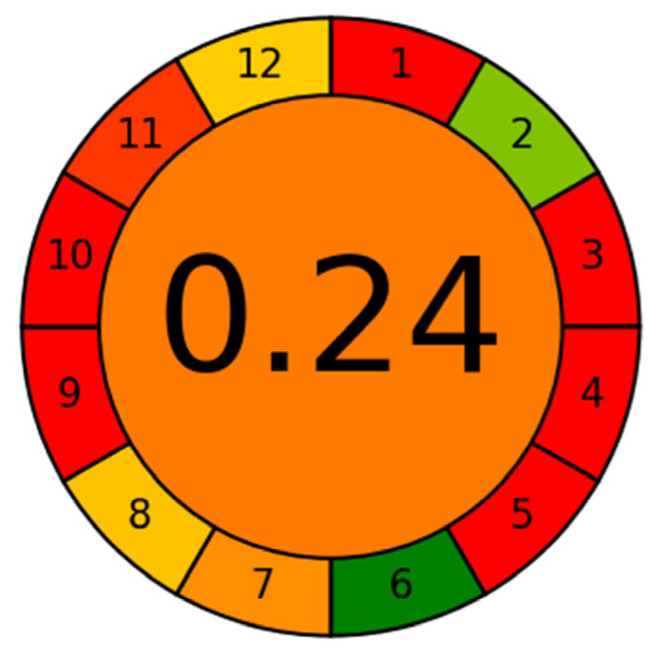
Greenness assessment score using the AGREE. The color scale represents the level of compliance with each principle of Green Analytical Chemistry, ranging from red (low greenness) to green (high greenness), corresponding to increasing AGREE scores from 0 to 1.

The resulting AGREE score was 0.24 (Figure 3), indicating relatively low level of environmental sustainability. This low score primarily reflects the use of toxic organic solvents (ACN, MeOH, and n-hexane) in the extraction and chromatographic steps as well as the reliance on LC-MS/MS instrumentation, which is energy-intensive. In addition, the QuEChERS-based workflow generates a notable amount of solid and liquid waste, including spent sorbents and solvent residues, further contributing to the environmental burden.

Nevertheless, several aspects of the method align with green analytical principles. The overall analysis time is relatively short (10 min per run), the method uses readily available reagents without isotope-labeled internal standards or specialized sorbents such as MIPs, and the sample amount required for analysis is modest, which reduces the overall consumption of reagents. These features make the method practical for routine monitoring and provide a basis for future improvements toward more sustainable analytical protocols.

### 2.7. Limitations and Future Perspectives

Although the developed LC-MS/MS method provides reliable quantification of methenamine in raw milk and bovine muscle, several limitations remain. The analyte’s inherent properties—its extremely high polarity and susceptibility to acid-catalyzed hydrolysis—restricted the selection of extraction solvents and chromatographic conditions. Despite the improved stability achieved under mildly basic extraction conditions, residual matrix components such as lipids, sugars, organic acids, and other polar co-extractives continued to contribute to matrix effects, requiring additional purification using n-hexane and PSA. Furthermore, the greenness evaluation indicated a low environmental sustainability (see Section 2.6), mainly due to the use of toxic organic solvents and the high energy consumption associated with LC-MS/MS analysis.

Future studies should aim to enhance both analytical performance and sustainability. Possible directions include investigating greener or lower-volume extraction solvents, exploring alternative sorbents to further reduce matrix interferences, and applying advanced chromatographic or MS acquisition strategies to improve selectivity and robustness. The development of miniaturized or solvent-free extraction techniques may also reduce environmental impact. Additionally, extending this method to other complex animal-derived matrices would broaden its applicability and practical relevance.

## 3. Materials and Methods

### 3.1. Reagents and Materials

Methenamine standard (purity 99.8%) was purchased from Sigma-Aldrich (St. Louis, MO, USA). HPLC-grade ACN and MeOH were obtained from J.T. Baker (Randor, PA, USA), and *n*-hexane was supplied by SK Chemicals (Seongnam, Republic of Korea). Ammonia water (28–30%, NH_3_ basis), anhydrous MgSO_4_, and Na_2_SO_4_ were purchased from Sigma-Aldrich, while PSA and C_18_ sorbents were obtained from Agilent Technologies (Santa Clara, CA, USA). LC-MS grade water, ACN, and formic acid, used as mobile phase solvents, were purchased from Fisher Chemicals (Pittsburgh, PA, USA). Ammonium acetate (purity > 98%) was obtained from Sigma-Aldrich (St. Louis, MO, USA), and 13 mm/0.2 µm PTFE filters were supplied by Whatman (Buckinghamshire, UK). A methenamine stock solution (100 mg/L) was prepared in MeOH, and working solutions (2 mg/L) were obtained by serial dilution with MeOH. All solutions were stored in amber tubes at −20 °C in the dark.

### 3.2. Analytical Procedure

#### 3.2.1. Sample Collection

A total of 32 raw milk samples were collected from milk collection centers located in seven regions across Korea. Within one week of collection, the samples were aliquoted into appropriate quantities and stored at −20 °C. In addition, 25 bovine muscle samples were collected from slaughter houses in eight regions. The muscle samples were homogenized and aliquoted, and stored under the same frozen conditions (−20 °C).

#### 3.2.2. Sample Pretreatment

An aliquot of 2 g of homogenized raw milk or bovine muscle was placed into a 50 mL centrifuge tube. A 10 mL mixture of ACN:MeOH (7:3, *v*/*v*) and 100 µL of ammonia water (28–30%, NH_3_ basis) were added, and the tube was vortexed for 10 min. After centrifugation at 4 °C and 5000× *g* for 10 min, the supernatant was transferred to a new tube and mixed with 10 mL of *n*-hexane. Following vortexing for 10 min and centrifugation under the same conditions, the upper hexane layer was removed. PSA (100 mg) was added to the remaining layer, vortexed for 5 min, and centrifuged again. The final extract was evaporated to 0.5 mL under a stream of nitrogen at 40 °C, reconstituted with 0.5 mL of ACN, centrifuged at 22,000× *g* for 10 min, and filtered through a 0.2 µm PTFE filter prior to LC-MS/MS analysis.

#### 3.2.3. HPLC-MS/MS Analysis

Chromatographic analysis was performed on a Sciex Exion HPLC system equipped with a Waters Xbridge HILIC (Waters Corporation, Milford, MA, USA) column (2.1 mm × 100 mm, 3.5 µm), as described in Section 2.2. The column temperature was maintained at 35 °C. The mobile phases consisted of 0.1% formic acid with 5 mM ammonium acetate in water (solvent A) and 100% ACN (solvent B). The flow rate was set at 0.2 mL/min, and the injection volume was 10 µL. The chromatographic system was operated in isocratic mode at an A:B ratio of 25:75 for 10 min.

Mass spectrometric analysis was carried out on a QTRAP 5500 mass spectrometer (AB Sciex, Framingham, MA, USA) equipped with an ESI source. Methenamine was analyzed in positive ESI mode under multiple reaction monitoring (MRM). The optimized ESI parameters were as follows: ion spray voltage, 5.5 kV; source temperature, 500 °C; curtain gas, 35 psi; nebulizer gas (Gas 1), 50 psi; and heater gas (Gas 2), 50 psi. Three precursor/product ion pairs were optimized for MRM, with collision energy and other compound-specific parameters adjusted accordingly. MRM transitions and their optimized conditions are shown in Table 4. Data acquisition and processing were performed using Analyst^®^ software (v1.7.1) and MultiQuant^TM^ (v3.0.3).

### 3.3. Validation of the Method

#### 3.3.1. Assessments of Linearity, LOD, LOQ, Accuracy and Precision

The analytical method developed in this study was validated in accordance with CODEX CAC/GL 71-2009 guidelines [30], including assessments of linearity, LOD, LOQ, accuracy, and precision. Linearity was evaluated from matrix-matched calibration curves prepared by adding standard solutions to pretreated blank matrices, with linear regression applied to the resulting peak area- concentration data. The calibration range was 0.4–100 ng/mL. The LOD and LOQ were determined using the standard deviation of the residuals (σ) and the slope (S) of the calibration curve, in accordance with Equation (2) described in Section 2.3.2.

Precision was assessed by repeated analysis of samples spiked at three concentration levels: 1, 10, and 40 μg/kg for raw milk, and 5, 10, and 40 μg/kg for bovine muscle. Intra-day repeatability was evaluated by six replicates per concentration on the same day, while inter-day repeatability was determined from twelve replicates per concentration over three consecutive days. Accuracy was determined from the recovery of methenamine in blank samples spiked at the same concentrations used for the precision evaluation.

#### 3.3.2. Stability Tests

Stability testing for methenamine was performed using QC samples prepared at low (5 ug/kg) and high concentration (40 ug/kg) levels. In routine practice, samples are typically stored at −20 °C and analyzed immediately after sample preparation. However, in cases where instrument issues occur, pretreated samples may be temporarily stored at −80 °C and analyzed once the issue has been resolved. Therefore, in this study, stability evaluation included short-term stability, freeze–thaw stability and autosampler stability. For short-term stability, samples were analyzed immediately after sample preparation at room temperature and compared with samples that had been at −80 °C for two days, thawed at room temperature, and subsequently re-analyzed. Freeze–thaw stability was assessed by subjecting fortified samples to three consecutive freeze–thaw cycles between −20 °C storage and complete thawing at room temperature. After the third cycle, QC samples were prepared and analyzed, and the results were compared with those obtained from freshly prepared samples. Autosampler stability was evaluated by maintaining final extracts in the LC-MS/MS autosampler at 10 °C for 24 h and comparing the results with those of the time-zero samples.

## 4. Conclusions

In this study, we developed and validated a rapid, reliable, and cost-effective LC-MS/MS method for the determination of methenamine in raw milk and bovine muscle. By optimizing the extraction solvent composition, fat removal process, and purification steps, the method achieved significantly higher recovery rates compared to the existing MFDS method, while maintaining low LOD and LOQ (0.16–0.21 μg/kg and 0.49–0.64 μg/kg, respectively). The proposed method meets the PLS criteria, exhibits excellent linearity (R^2^ > 0.999), accuracy (78.1–102.8%), and precision (CV ≤ 8.75%), and effectively minimizes matrix effects through matrix-matched calibration. Application to incurred samples demonstrated its suitability for routine monitoring; methenamine was detected in 6.25% of raw milk samples at levels below the PLS threshold, while no residues detected in bovine muscle. Given its simplicity, use of readily available reagents, and high analytical performance, this method offers substantial practical value for regulatory surveillance and food safety management of veterinary drug residues in livestock-derived products.

## Figures and Tables

**Table 1:** Comparison of proposed method and MFDS method.

Method	1 (Proposed Method)	2 (MFDS Method)
Sample	2 g of samples	5 g of samples
Extraction	mixture of ACN and MeOH (ACN:MeOH = 7:3, *v*/*v*) (10 mL) ammonia water (100 µL)	10 mM Ammonium formate in ACN (20 mL)
Purification	Hexane (10 mL) PSA (100 mg)	Hexane (20 mL) MgSO_4_ (900 mg) PSA (50 mg)
Evaporation	Under N_2_(g), 40 °C until 0.5 mL of extracts	
Reconstitution	ACN 0.5 mL	ACN 1.5 mL
Mobile phase A	0.1% formic acid and 5 mM ammonium acetate in water	10 mM ammonium formate in water

MFDS: the Ministry of Food and Drug Safety in Korea, ACN: acetonitrile, MeOH: methanol, PSA: primary secondary amine, MgSO_4_: magnesium sulfate.

**Table 2:** Recovery and precision of methenamine in raw milk and bovine muscle at three spiked levels.

	Spiked Concentration (µg/kg)	Intra-Day (*n* = 6)		Inter-Day (*n* = 12)	
		Recovery (%)	CV (%)	Recovery (%)	CV (%)
raw milk	1	95.4	5.94	96.0	7.10
	10	102.8	7.64	101.6	5.97
	40	95.8	3.13	95.9	4.59
bovine muscle	5	91.5	3.82	91.4	7.74
	10	78.1	3.57	83.5	8.75
	40	94.3	4.30	91.5	7.03

CV: coefficients of variation.

**Table 3:** Accuracy (mean ± CV%, n = 3) and relative error (%) of methenamine under various stability conditions.

Matrix	QC	Room Temp (Time-Zero)	Short Term Freezer −80 °C (2 d)	Freeze–Thaw (3 Cycles)	Autosampler 24 h
Raw milk	Low QC	95.34 ± 4.44	93.31 ± 3.22 (−2.13)	97.41 ± 3.78 (2.17)	91.58 ± 4.98 (−3.94)
	High QC	89.90 ± 3.69	88.37 ± 5.21 (−1.70)	95.92 ± 3.11 (6.70)	87.66 ± 10.52 (−2.49)
Bovine muscle	Low QC	98.56 ± 3.36	102.20 ± 2.28 (3.69)	98.07 ± 0.99 (−0.50)	95.40 ± 3.28 (−3.21)
	High QC	91.51 ± 4.70	94.75 ± 4.05 (3.54)	85.67 ± 4.77 (−6.38)	94.26 ± 5.22 (3.01)

Values in parentheses indicate the relative error (%), calculated relative to the room temperature (time-zero) QC samples using $Relative\;error\left(\%\right)=\frac{Measured\;value-Time\text{-}zero\;value}{Time\text{-}zero\;value}\times 100$.

**Table 4:** Optimized MS/MS parameters for methenamine.

Precursor Ion (*m*/*z*)	Product Ion (*m*/*z*)	Dwell Time (ms)	Retention Time (min)	DP (Volts)	EP (Volts)	CE (Volts)	CXP (Volts)
140.9	112.1	100	3.71	136	10	19	12
	85.1					25	14
	98					19	18

DP: declustering potential, EP: entrance potential, CE: collision energy, CXP: cell exit potential.

## Data Availability

The original contributions presented in this study are included in the article/Supplementary Materials. Further inquiries can be directed to the corresponding author.

## Supplementary Materials

The following supporting information can be downloaded at: https://www.mdpi.com/article/10.3390/molecules30244807/s1, Figure S1: Full LC-MS/MS chromatograms of all sample types analyzed in this study; Figure S2: Calibration curves for methenamine in (A) raw milk and (B) bovine muscle over the concentration range of 0.4–100 µg/kg.
