# Supplementary figures and images for: Development and Validation of an LC-MS/MS Method for the Quantification of Methenamine in Raw Milk and Bovine Muscle and Its Application to Incurred Samples
Source: Molecules. 2025 Dec 17;30(24):4807. doi: 10.3390/molecules30244807 (PMC12735475; doi:10.3390/molecules30244807)

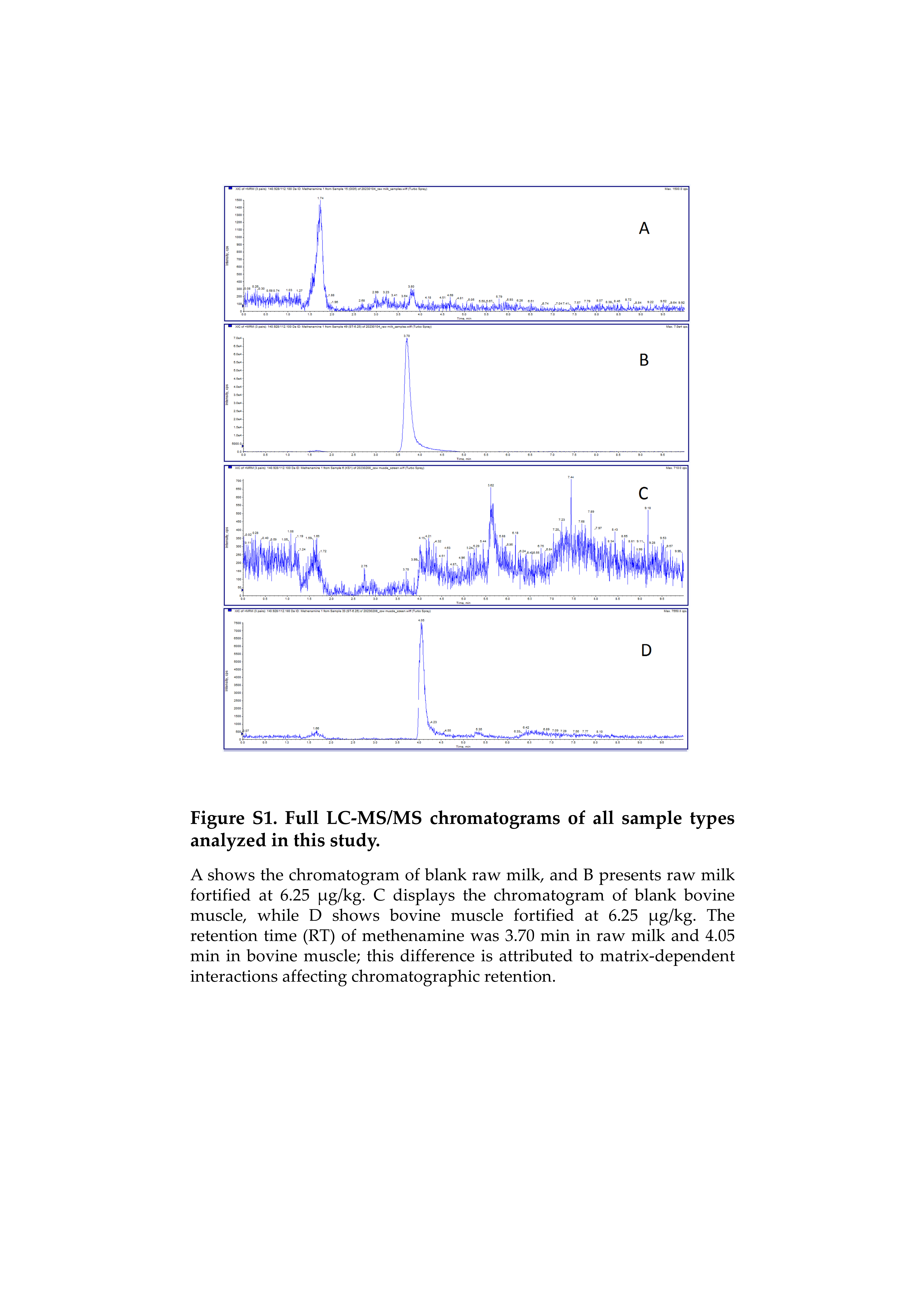

Supplement: Supplementary file 1 [file molecules-30-04807-s001.zip › Supplementary materials/Figure S1.tif]

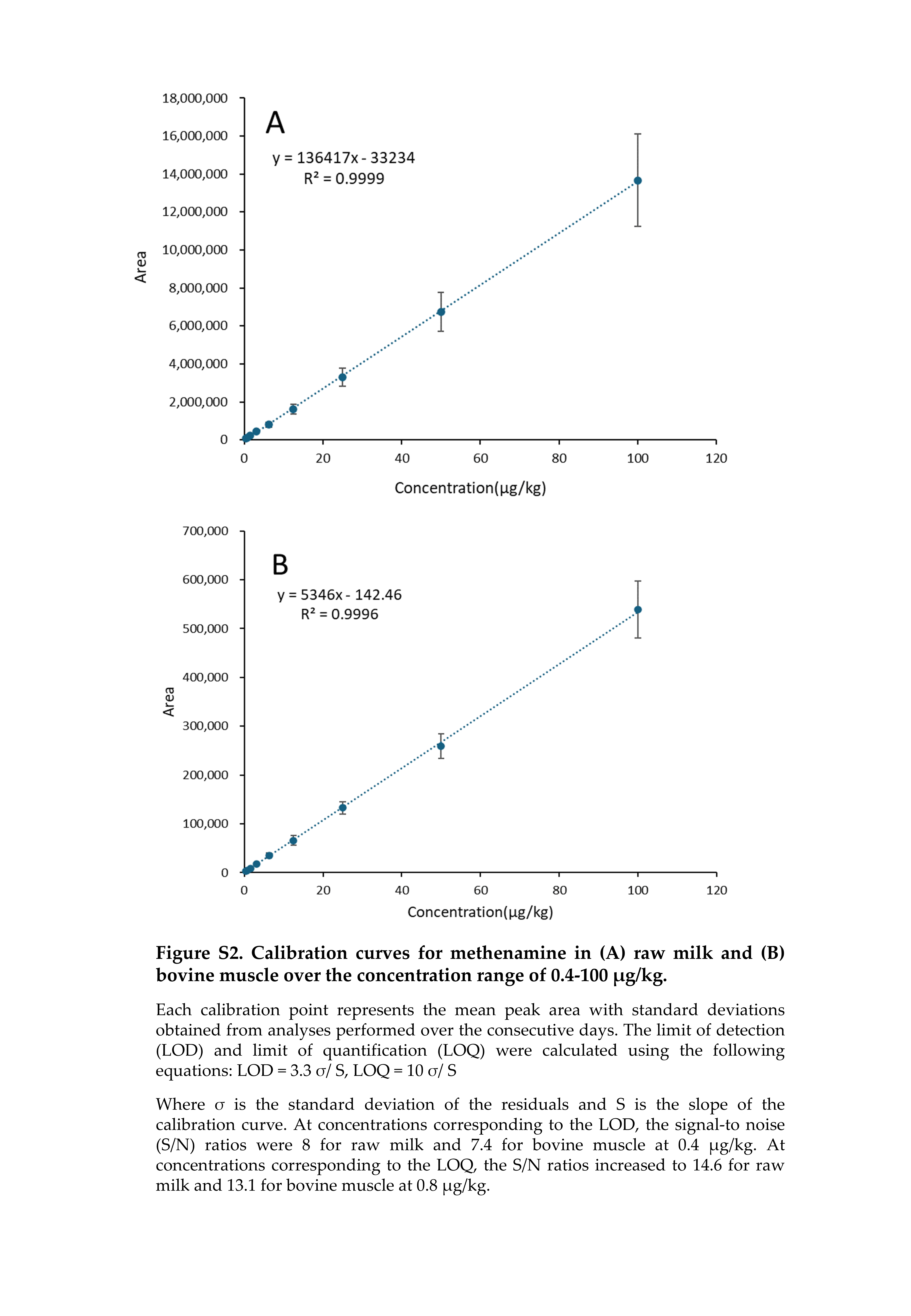

Supplement: Supplementary file 1 [file molecules-30-04807-s001.zip › Supplementary materials/Figure S2.tif]
